# Cry1Ac Mixed with Gentamicin Influences the Intestinal Microbial Diversity and Community Composition of Pink Bollworms

**DOI:** 10.3390/life14010058

**Published:** 2023-12-28

**Authors:** Zhan-Bin Sun, Ya-Feng Hu, Han-Jian Song, Sheng-Bo Cong, Ling Wang

**Affiliations:** 1 China Food Flavor and Nutrition Health Innovation Center, Beijing Technology and Business University, Beijing 100048, China; 2 Key Laboratory of Integrated Pest Management on Crops in Central China, Ministry of Agriculture and Rural Affairs, Hubei Key Laboratory of Crop Disease, Insect Pests and Weeds Control, Institute of Plant Protection and Soil Science, Hubei Academy of Agricultural Sciences, Wuhan 430064, China

**Keywords:** alpha diversity, PCoA analysis, LEfSe analysis, Bt resistance, intestinal microorganisms, Illumina MiSeq sequencing and analysis

## Abstract

Pink bollworms severely affect the production of cotton. The method currently used for pink bollworm control is the planting of Bt (*Bacillus thuringiensis*) protein-expressing transgenic cotton. However, pink bollworms can develop strong resistance to Bt proteins in transgenic cotton because of the large planting area and long planting time of this crop, which severely affects the control of pink bollworms. Intestinal microorganisms play very important roles in insect growth, development and Bt resistance. However, the effect of intestinal microorganisms on pink bollworm Bt resistance is still unclear. The current study aimed to analyze the effect of intestinal microorganisms on the Bt resistance of pink bollworms. Intestinal microorganisms associated with Bt resistance were initially screened through Illumina MiSeq sequencing and analysis. The results showed that feeding with a mixture of gentamicin, Cry1Ac and an artificial diet could significantly increase the mortality of pink bollworm larvae compared with feeding with of a mixture of Cry1Ac and an artificial diet or an artificial diet alone. The microbial diversity, community structure and composition of the pink bollworm larval intestine were significantly influenced by feeding with a mixture of gentamicin, Cry1Ac and an artificial diet. Several intestinal bacteria with significantly altered abundances after treatment with gentamicin were preliminarily screened as potential resources for addressing Bt toxicity. This study provides useful strategies for addressing the Bt resistance of pink bollworms.

## 1. Introduction

The pink bollworm (*Pectinophora gossypiella*) can severely affect the production of cotton. The larvae of pink bollworms mainly infect the flower buds and bells of cotton plants and cause large yield reductions and economic losses each year [1,2]. The commonly used methods of controlling pink bollworms are chemical control and the planting of Bt (*Bacillus thuringiensis*) protein-expressing transgenic cotton. Among these control methods, the application of Bt protein-expressing transgenic cotton is widely used because it can effectively control the population of pink bollworms [3,4].

Pink bollworms can easily generate strong resistance to Bt proteins in transgenic cotton because of the large planting area and long planting duration for this crop, which severely affects the control effect of Bt protein-expressing transgenic cotton against pink bollworms. There are numerous factors that can influence insect resistance to Bt proteins, including alterations in the activities of insect midgut proteases, changes in the special receptors of the insect midgut and changes in other physiological and biochemical factors [5,6,7]. The most common strategies to prevent agricultural pests from developing resistance to Bt proteins include “high dosage/sanctuary” or gene superposition, which enhances the activity of the Cry protein via combination with other proteins or the modification of Cry genes [8,9,10,11]. Besides the above strategies, insect intestinal microorganisms have been reported to be involved in Bt protein resistance [12,13,14,15].

The insect intestine contains abundant microbial resources, which play very important roles in insect growth and development and other functions [12,13]. Several studies have shown that intestinal microorganisms can also influence the resistance of insects to Bt proteins. Khainga et al. [14] found that the intestinal microbes of cotton bollworms were significantly altered after treatment with antibiotics. An in-depth study found that the larval mortalities of cotton bollworms were markedly reduced by treatments with Cry1Ac after being pretreated with antibiotics, and the mechanism might involve antibiotics inhibiting the growth of some intestinal microorganisms, therefore influencing the toxicity of Bt to cotton bollworms. Visweshwar et al. [15] suggested that intestinal microbes have a significant effect on the toxicity of Cry1Ac to cotton bollworm larvae. Tao [16] investigated the influence of different intestinal microorganisms on the Bt resistance of *Plutella xylostella* and found that different types of microbes exhibited different effects on the Bt resistance of *P. xylostella*. The mechanism by which intestinal microorganisms affect insect Bt resistance might be due to these microbes influencing the activation of Bt protoxin and the degradation of the Bt proteins or the toxicity of Bt to insects [17,18]. However, until now, studies have seldom reported the effect of intestinal microorganisms on pink bollworm Bt resistance [19].

To investigate the influence of intestinal microorganisms on pink bollworm Bt resistance, in the current study, antibiotics were used to treat pink bollworm larvae, and the effect of microbes on Bt resistance was analyzed. Then, the correlation between intestinal microbes and Bt resistance was determined, and microbes associated with potential Bt resistance were initially screened. This study has significance for further understanding the mechanism of pink bollworm Bt resistance.

## 2. Materials and Methods

### 2.1. Pink Bollworms

The pink bollworm-sensitive type strain QJ-S was collected from Qianjiang City, Hubei Province, China. QJ-S was fed an artificial diet for more than 10 years (an average of one generation per month and over 120 generations in total) and was not treated with any insecticides or Cry1Ac (Bt resistance proteins). QJ-S larvae were fed at 28 ± 1 °C with a photoperiod and relative humidity of 16:8 (L:D) and 50 ± 10%, respectively. Cry1Ac was purchased from Zhongbao Biotechnology Company, Beijing, China.

### 2.2. Screening of Antibiotics Associated with Cry1Ac Resistance

In total, three antibiotics with different bacterial inhibition spectra, including gentamicin, rifampicin and ampicillin, were used in this study. All three antibiotics were used at four gradient concentrations per insect: 60, 120, 240 and 480 μg/mL. The Cry1Ac concentration was adjusted to 0.12 μg/mL.

The diet incorporation method was used to screen antibiotics associated with Cry1Ac resistance in pink bollworms. Cry1Ac, together with different concentrations of antibiotics, was mixed into the artificial diet and then fed to the larvae of QJ-S. QJ-S larvae were fed the artificial diet alone, the artificial diet mixed with Cry1Ac and the artificial diet mixed with gentamicin as controls. Finally, the mortality associated with each treatment was calculated to evaluate the effects of each antibiotic. Each replication treatment included 24 pink bollworms. Four replications were used for each treatment.

The SAS 9.1.3 software (SAS Institute Inc., Cary, NC, USA) was used to analyze the differences in mortality between all treatments with Duncan’s multiple range test. A *p* lower than 0.05 was regarded as indicating a significant difference.

### 2.3. Experimental Design

Three treatments were used to analyze the intestinal microorganisms from QJ-S pink bollworms that were associated with Cry1Ac resistance. The CK group contained QJ-S larvae fed the artificial diet; the Bt group contained QJ-S larvae fed the artificial diet and 0.12 μg/mL of Cry1Ac; the Gen group contained QJ-S larvae fed the artificial diet, 0.12 μg/mL of Cry1Ac and 60 μg/mL of gentamicin. Each treatment was replicated four times.

### 2.4. Intestinal DNA Extraction and PCR Amplification

The intestines of pink bollworm larvae from each treatment were obtained as previously described. Then, intestinal DNA was extracted using a FastDNA Spin Kit (MP, Santa Ana, CA, USA) according to the manufacturer’s instructions. The integrity, quality and concentration of the extracted DNA were detected via 1% agarose gel electrophoresis and a NanoDrop2000 (Thermo Fisher Scientific, Waltham, MA, USA).

The primer pair 338F (5′-ACTCCTACGGGAGGCAGCAG-3′) and 806R (5′-GGACTACHVGGGTWTCTAAT-3′) was used to amplify the V3-V4 region of the 16S rDNA of intestinal bacteria [20]. The 20 μL PCR system consisted of 10 ng/μL of template DNA; 10 μL of 2 × Pro Taq; 0.8 μL of the 338F and 806R primers; and ddH_2_O. The PCR was conducted with an ABI GeneAmp^®^ 9700 (Applied Biosystems, Foster City, CA, USA) with the following program: predenaturation at 95 °C for 3 min; 30 cycles of denaturation at 95 °C for 30 s, annealing at 55 °C for 30 s and extension at 72 °C for 45 s; and a final extension at 72 °C for 10 min. The PCR products of all the treatments were detected through 2% agarose gel electrophoresis and then recovered with an AxyPrep DNA Gel Extraction Kit.

### 2.5. Illumina MiSeq Library Construction and Sequencing

Illumina MiSeq libraries for all the treatments were constructed using the TruSeqTM DNA Sample Prep Kit according to the manufacturer’s protocols. Illumina MiSeq sequencing was performed by Majorbio Co., Ltd. (Shanghai, China). The PE raw reads obtained from Illumina MiSeq sequencing were assembled according to overlap relationships using the Flash 1.2.11 software, and low-quality reads were filtered as previously reported [21,22,23]. Operational taxonomic units (OTUs) are the basic units for bioinformatic analysis and were clustered and sequenced with 97% similarity using the Uparse 7.0.1090 software [24]. The taxonomy of the OTUs was analyzed according to the RDP classifier at 97% similarity [25]. All sequenced data were uploaded to NCBI SRA (National Center for Biotechnology Information Sequence Read Archive) to obtain accession numbers.

### 2.6. Bioinformatic Analysis

Alpha diversity analysis was used to reflect changes in microbial diversity and richness among the different treatments. Among the alpha diversity indices, the Shannon and Simpson indices were used to indicate the microbial diversity, and the Sobs, Ace and Chao indices were used to reflect the microbial richness. PCoA was used to analyze the differences in microbial community composition between the different treatments. Microbial community composition analysis was conducted to reflect the composition of the microbial community at the phylum, class and genus taxonomic levels in each treatment, as well as the relative abundance of beneficial microorganisms. The Kruskal–Wallis H test was conducted to assess the difference in abundance of genera between the different treatments, and a *p*-value lower than 0.05 was regarded as indicating a significant difference. Linear discriminant analysis effect size (LEfSe) analysis was performed with a linear discriminant analysis (LDA) score higher than 2, which was used to identify differences in bacterial abundance between different treatments [26].

## 3. Results

### 3.1. Screening of Antibiotics

Feeding with Cry1Ac significantly increased the mortality of QJ-S compared with feeding with the artificial diet alone (Table 1). Adding three different types of antibiotics showed that rifampicin and ampicillin treatment had no marked effect on the mortality of QJ-S. Mixing an artificial diet and Cry1Ac with gentamicin significantly increased the mortality of QJ-S compared with feeding with the artificial diet and Cry1Ac (Table 1). However, there was no significant difference in the mortality of QJ-S between the four concentrations (Table 1). Moreover, feeding with a mixture of gentamicin and an artificial diet had no significant effect on the mortality of QJ-S compared with feeding with the artificial diet alone (Table 1). Therefore, we selected gentamicin at a concentration of 60 μg/mL for the subsequent experiments.

### 3.2. Alpha Diversity Analysis

**Figure 1:**
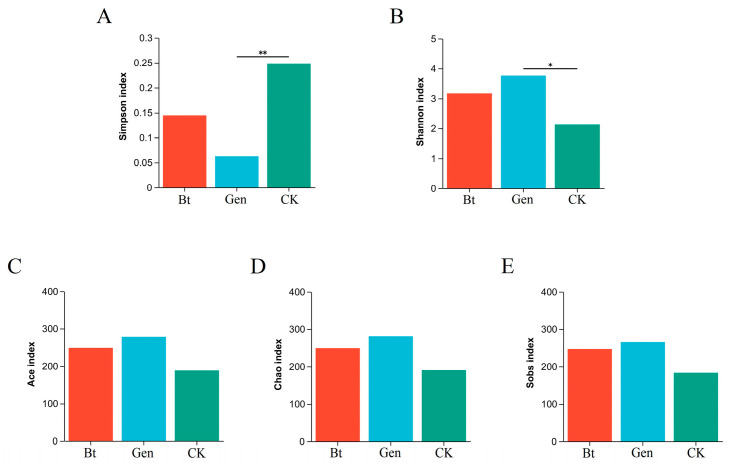
Alpha diversity analysis per genus level. The *x*-axis represents different treatments; the *y*-axis represents different indices. (**A**) Simpson index, (**B**) Shannon index, (**C**) Ace index, (**D**) Chao index, (**E**) Sobs index. Bt, feeding with artificial diet and Cry1Ac; Gen, feeding with 60 μg/mL of gentamicin, artificial diet and Cry1Ac; CK, feeding with artificial diet. * *p* < 0.05, ** *p* < 0.01.

The Shannon and Simpson values in the Bt group were not significantly altered compared with those in the CK group, which indicated that 0.12 μg/mL of Cry1Ac could not influence the microbial diversity of pink bollworm larvae (Figure 1A,B). However, the values of the Shannon and Simpson indices in the Gen group were significantly higher and lower than those in the CK group, respectively, which indicated that gentamicin could markedly affect the microbial diversity of pink bollworm larvae. The microbial richness indices, including Ace, Sobs and Chao1, were not markedly altered among the three groups, which indicated that the use of 0.12 μg/mL of Cry1Ac or gentamicin could not change the microbial richness of pink bollworm larvae (Figure 1C–E).

### 3.3. PCoA Analysis

**Figure 2:**
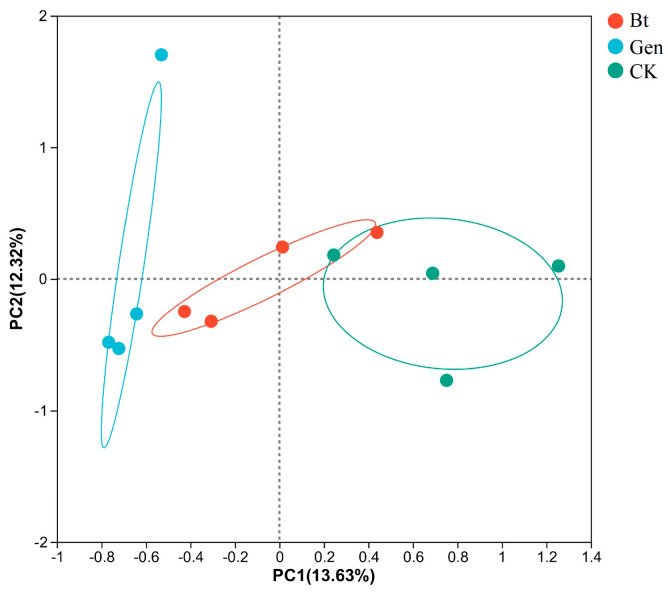
Principal coordinate analysis. The *x*-axis and *y*-axis represent variance in the community structures of intestinal bacteria. Bt, feeding with artificial diet and Cry1Ac; Gen, feeding with 60 μg/mL of gentamicin, artificial diet and Cry1Ac; CK, feeding with artificial diet.

A PC1 value of 13.63% and a PC2 value of 12.32% represent variability among the three different treatments. PCoA showed that the Gen group was separated from the other two groups, which indicated that the microbial community of pink bollworm larvae was significantly altered after treatment with gentamicin. However, the Bt group was not separated from the CK group, which demonstrated that the addition of 0.12 μg/mL of Cry1Ac had no marked effect on the microbial community of pink bollworm larvae (Figure 2).

### 3.4. Microbial Community Composition Analysis

The microbial community compositions among the three treatments were analyzed at three taxonomic levels (phylum, class and genus).

The dominant phyla in the three groups were the same but with different abundances. The three most abundant phyla in the CK, Bt and Gen groups were Bacillota (83.24% in CK, 54.78% in Bt, 39.71% in Gen), Pseudomonadota (8.14% in CK, 25.47% in Bt, 21.71% in Gen) and Actinomycetota (6.80% in CK, 7.81% in Bt, 19.99% in Gen) (Figures S1A and S2A).

The dominant classes in the CK and Gen groups were similar, with the three most abundant classes in the CK and Gen groups being Bacilli (82.48% in CK, 38.25% in Gen), Actinobacteria (6.63% in CK, 15.73% in Gen) and Gammaproteobacteria (6.04% in CK, 12.65% in Gen). The dominant classes in the Bt group were slightly different from those in the other two groups, namely, Bacilli (53.61%), Gammaproteobacteria (18.66%), Alphaproteobacteria (6.80%) and Actinobacteria (6.64%) (Figures S1B and S2B).

At the genus level, the dominant genera among the three groups were quite different. In the CK group, the top three genera were *Lactococcus* (45.26%), *Streptococcus* (14.91%) and *Lactobacillus* (9.29%). In the Bt group, the top three genera were *Lactococcus* (45.26%), *Burkholderia–Caballeronia–Paraburkholderia* (8.55%) and *Lactobacillus* (8.52%). In the Gen group, the top three genera were *Lactococcus* (12.54%), *Burkholderia–Caballeronia–Paraburkholderia* (7.09%) and *Acidothermus* (8.52%) (Figures S1C and S2C).

**Figure 3:**
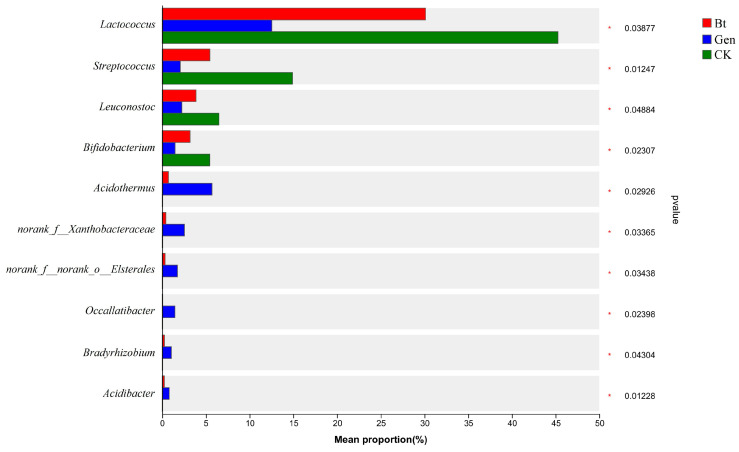
Community abundance analysis with different treatments. The *x*-axis indicates the proportion of community abundance; the *y*-axis represents different bacterial genera. Bt, feeding with artificial diet and Cry1Ac; Gen, feeding with 60 μg/mL of gentamicin, artificial diet and Cry1Ac; CK, feeding with artificial diet. * *p* < 0.05.

This in-depth investigation of the changes in the abundance of bacteria among the three groups was helpful for screening bacteria associated with Cry1Ac resistance. The abundances of numerous bacterial genera were significantly altered after treatment with 0.12 μg/mL of Cry1Ac or gentamicin. The abundances of genera such as *Lactococcus*, *Streptococcus*, *Leuconostoc* and *Bifidobacterium* were significantly reduced after feeding with 0.12 μg/mL of Cry1Ac compared with the artificial diet, and the abundances of the four genera continued to decrease significantly after additional feeding with gentamicin (Figure 3). The abundances of other genera, such as *Acidothermus*, *Bradyrhizobium* and *Acidibacter*, exhibited the opposite trend to those of the above genera. Their abundances continued to increase significantly from the CK and Bt groups to the Gen group (Figure 3).

### 3.5. LEfSe Analysis

LEfSe analysis showed that bacteria in all three treatments were significantly different from the phylum to genus taxonomic levels. The bacteria in the CK group included two orders, three families and five genera. The bacteria in the Bt group included two orders, three families and three genera. However, the number of significant bacteria in the Gen group was higher than in the other two groups. The bacteria in the Gen group included 8 phyla, 8 classes, 17 orders, 17 families and 17 genera (Figure S3).

## 4. Discussion

The development of Bt resistance by pink bollworms is an important factor that severely affects the survival of pink bollworms. This study provides important insights into seeking effective and inexpensive methods to address pink bollworm Bt resistance. Previous studies have reported that, in other lepidopteran insects, such as cotton bollworms, intestinal microorganisms are closely related to the Bt resistance of cotton bollworms [27,28]. However, the correlation between intestinal microorganisms and pink bollworm Bt resistance has not been studied. Therefore, to investigate the relationships between intestinal microorganisms and Bt resistance in pink bollworms and to screen potential intestinal microbial resources related to Bt resistance, in this study, pink bollworm larvae were fed Cry1Ac and gentamicin. The results showed that the mortality of pink bollworm larvae increased significantly after feeding with gentamicin. An in-depth analysis revealed that the gentamicin treatment significantly altered the intestinal microbial diversity and community composition. Microorganisms related to Bt resistance were also preliminarily screened. This study provides new insights into the Bt resistance of pink bollworms.

In this study, the intestinal microbial diversity and community composition of pink bollworms were not significantly altered in the group fed an artificial diet and 0.12 μg/mL of Cry1Ac compared with the group fed only an artificial diet. This might be due to the use of low-dosage Cry1Ac in this study. The reasons for using low-dose Cry1Ac were as follows: we needed to ensure that the survival rate of pink bollworm larvae was sufficient to obtain material for Illumina MiSeq sequencing after feeding with gentamicin mixed with Cry1Ac. If the concentration of Cry1Ac used in this study had been too high, sufficient pink bollworm larvae for the subsequent experiments would not have been obtained after treatment with gentamicin. Therefore, using a low dosage of Cry1AC could help us to establish the relationship between the intestinal microbial community and pink bollworm Bt resistance.

We used three antibiotics with different inhibition spectra in this study, mainly aiming to screen more bacterial resources associated with Bt resistance. We expected the antibiotics used in this study to have two effects. Firstly, the antibiotics would not influence the mortality of pink bollworm larvae because, if the antibiotics used in this study had an impact on the mortality of pink bollworm larvae, we would not have been able to analyze the increased mortality of pink bollworm larvae in the treatment involving feeding with the mixture Bt protein, antibiotics and artificial diet; this was due to antibiotics eliminating microorganisms associated with Bt protein degradation in pink bollworm intestines. Secondly, the mortality of pink bollworm larvae fed with a mixture of Bt protein and antibiotic treatments was significantly different compared with feeding them Bt protein only. Antibiotics might eliminate microorganisms that are associated with Bt protein degradation; hence, the mortality of pink bollworm larvae would be significantly higher in Bt protein and antibiotic treatments compared with feeding with Bt protein only. Similar phenomena have been found in cotton bollworms and *Plutella xylostella* [14,16]. In this study, the results showed that only treatment with gentamicin, incorporated with Cry1Ac and an artificial diet could significantly increase the mortality of pink bollworm larvae, as the mortality was not markedly altered after feeding with rifampicin and ampicillin and a mixture of Cry1Ac and an artificial diet. Therefore, we did not use a cocktail of three antibiotics in this study for the analysis of intestinal microorganisms.

Four gradient concentrations of gentamicin, 60, 120, 240 and 480 μg/mL, were used to measure the mortality of pink bollworm larvae. We found the mortality of pink bollworm larvae had no significant difference between the four concentrations of gentamicin. Based on economic considerations, we selected 60 μg/mL of gentamicin for subsequent experiments. In addition, we did not select higher concentrations of antibiotics such as 960 μg/mL to evaluate the antibiotic dose effect on insect mortality for the following reasons. The objective of this research was mainly to explore potential intestinal microorganisms that are associated with Bt protein degradation. In this study, we found that pink bollworm mortality was significantly higher in mixed feeding with Bt protein and gentamicin compared with feeding them a Bt protein-only treatment. Thus, we could further investigate and preliminarily screen the intestinal microorganisms the species of which were dramatically altered after feeding them Bt protein and gentamicin compared with feeding Bt protein only. Therefore, the main role of antibiotics in this study was eliminating intestinal microorganisms in pink bollworms. Hence, we did not investigate the antibiotic dose that could have an effect on insect mortality.

In this study, we used three treatments for pink bollworm larvae—feeding with an artificial diet only; Cry1Ac mixed with an artificial diet; and a mixture of Cry1Ac, an artificial diet and gentamicin—to conduct intestinal microbial diversity and community composition analysis through Illumina Miseq sequencing. However, we did not feed pink bollworm larvae mixtures of gentamicin and an artificial diet as a treatment. This was mainly because, after investigating the role of gentamicin in the mortality of pink bollworm larvae, we found that, compared with feeding them with an artificial diet only, feeding with a mix of gentamicin and artificial diet did not significantly increase the mortality of pink bollworm larvae, which indicated that the addition of gentamicin did not influence the mortality of pink bollworm larvae. Therefore, the main role of gentamicin in this study was eliminating intestinal microbes in pink bollworms. The objective of this study was to analyze Bt protein-degradation-related intestinal microbes through a comparison of the microbial community composition between the Bt and Gen groups. Compared with the Bt group (feeding a mix of Cry1Ac and an artificial diet), the effect of gentamicin on intestinal microbial diversity and microbial community composition was the same as on the Gen group (feeding with a mixture of Cry1Ac, an artificial diet and gentamicin). Therefore, we did not feed pink bollworm larvae a mix of gentamicin and an artificial diet as a treatment to conduct Illumina Miseq sequencing.

Establishing the relationship between antibiotic treatments and Bt toxicity to pink bollworms is very important for screening potential microbial resources associated with Bt resistance. Similar approaches have been used to analyze the relevance of gut microbes and Bt resistance in insects such as cotton bollworms, mosquitos and gypsy moths [29,30,31]. In this study, the mortality of pink bollworms increased significantly after treatment with gentamicin, which indicated that gentamicin might kill or inhibit intestinal microorganisms associated with degrading or suppressing Cry1Ac. Therefore, the decrease in the abundance of intestinal microorganisms after treatment with gentamicin mixed with Cry1Ac compared with Cry1Ac alone might be correlated with Cry1Ac degradation or suppression. In our study, we found that the abundances of bacteria such as *Enterococcus* were reduced after treatment with gentamicin. *Enterococcus* species have been reported to limit the toxicity of Bt proteins [32]. Moreover, several bacteria, including the *Lactococcus*, *Leuconostoc* and *Bifidobacterium* species, have not previously been reported to be involved in inhibiting or degrading Bt proteins. However, their abundance was significantly reduced after feeding with gentamicin, indicating that they had the potential to inhibit the toxicity of Bt proteins, which could be further verified.

After feeding with the mixture of gentamicin, Cry1Ac and an artificial diet, the abundance of many bacteria, such as the *Acidothermus*, *Bradyrhizobium* and *Acidibacter* species were markedly increased compared with feeding a mixed Cry1Ac and artificial diet. The underlying reason might be due to competition between intestinal microorganisms. They have very complex relationships in the intestinal environment, both competitive and symbiotic. The abundance of some bacteria was very low because of a competitive limitation from other bacteria fed with an artificial diet or Bt protein. After treatment with gentamicin, those bacteria with competitive advantages in the intestine were killed or inhibited; therefore, the abundance of other low-abundance bacteria increased significantly. The roles of these bacteria in inhibiting the toxicity of Bt proteins need to be further studied.

Future work should focus on the screening, identification and application of microbial resources that affect Bt toxicity in pink bollworm larvae. Objective microbial resources could be obtained by detecting the influence of microbial resources on the activity or toxicity of Bt proteins in vitro. Then, the microbial resources could be used to reinoculate microbe-free intestines or to increase the microbial abundance of the regulatory intestines of pink bollworms and, thus, investigate the influence of microbial resources on the Bt resistance of pink bollworms. Bt protein-degradation-related intestinal microorganisms could be added to the diets of pink bollworms for feeding and further improve the Bt resistance of pink bollworms. This study provides useful strategies for addressing the Bt resistance of pink bollworms.

## 5. Conclusions

Feeding with Cry1Ac significantly increased the mortality of pink bollworm larvae compared with feeding them with an artificial diet only. Mixing with gentamicin further significantly increased the mortality of pink bollworm larvae. An in-depth study showed that mixing gentamicin and Cry1Ac markedly affected the microbial diversity, microbial community structure and microbial composition of the pink bollworm’s larval intestine.

## Figures and Tables

**Table 1:** Effect of antibiotics on pink bollworm survival.

Treatments (Feeding with)	Survival Numbers
Artificial diet	24 ^A^
Artificial diet and Cry1Ac	18 ^BC^
Artificial diet and Gentamicin (480 μg/mL)	24 ^A^
Artificial diet and Gentamicin (240 μg/mL)	24 ^A^
Artificial diet and Gentamicin (120 μg/mL)	24 ^A^
Artificial diet and Gentamicin (60 μg/mL)	24 ^A^
Rifampicin (480 μg/mL), artificial diet and Cry1Ac	16 ^CD^
Rifampicin (240 μg/mL), artificial diet and Cry1Ac	19 ^ABC^
Rifampicin (120 μg/mL), artificial diet and Cry1Ac	18 ^BC^
Rifampicin (60 μg/mL), artificial diet and Cry1Ac	21 ^AB^
Gentamicin (480 μg/mL), artificial diet and Cry1Ac	9 ^FG^
Gentamicin (240 μg/mL), artificial diet and Cry1Ac	9 ^FG^
Gentamicin (120 μg/mL), artificial diet and Cry1Ac	10 ^EFG^
Gentamicin (60 μg/mL), artificial diet and Cry1Ac	10 ^EFG^
Ampicillin (480 μg/mL), artificial diet and Cry1Ac	22 ^AB^
Ampicillin (240 μg/mL), artificial diet and Cry1Ac	22 ^AB^
Ampicillin (120 μg/mL), artificial diet and Cry1Ac	22 ^AB^
Ampicillin (60 μg/mL), artificial diet and Cry1Ac	22 ^AB^

Notes: The concentration of Cry1Ac is 0.12 μg/mL. In total, 24 pink bollworms were used for each replication per treatment. Different characters in the second column indicate a significant difference (*p* < 0.05).

## Data Availability

All sequenced data were submitted to the SRA database and obtained the following accession numbers: SRR26836624, SRR26836625, SRR26836586, SRR26836960, SRR26836884, SRR26836883, SRR26836961, SRR26836893, SRR26836892, SRR26836894, SRR26836895.

## Supplementary Materials

The following supporting information can be downloaded at https://www.mdpi.com/article/10.3390/life14010058/s1: Figure S1: Community composition of intestinal bacteria under different treatments.; Figure S2: Heatmap analysis under different treatments. x-axis means different treatments; Figure S3: Linear discriminant analysis effect size analysis of the community composition of intestinal bacteria in five taxon units under different treatments.
