# Supplementary material for: Cry1Ac Mixed with Gentamicin Influences the Intestinal Microbial Diversity and Community Composition of Pink Bollworms
Source: Life (Basel). 2023 Dec 28;14(1):58. doi: 10.3390/life14010058 (PMC10820413; doi:10.3390/life14010058)
Supplement: Supplementary file 1 [file life-14-00058-s001.zip › life-2766831-Supplementary files.pdf]

Supplementary

Cry1Ac Mixed with Gentamicin Influences the Intestinal Microbial Diversity and Community Composition of Pink Bollworms

Zhan-Bin Sun [1,*], Ya-Feng Hu [1], Han-Jian Song [1], Sheng-Bo Cong [2] and Ling Wang [2,*]

[1] China Food Flavor and Nutrition Health Innovation Center, Beijing Technology and Business University, Beijing 100048, China; 2130032039@st.btbu.edu.cn (Y.-F.H.); 2230301020@st.btbu.edu.cn (H.-J.S.)
[2] Key Laboratory of Integrated Pest Management on Crops in Central China, Ministry of Agriculture and Rural Affairs, Hubei Key Laboratory of Crop Disease, Insect Pests and Weeds Control, Institute of Plant Protection and Soil Science, Hubei Academy of Agricultural Sciences, Wuhan 430064, China; congshengbo@163.com
* Correspondence: twins5616@126.com (Z.-B.S.); wanglin20504@163.com (L.W.)

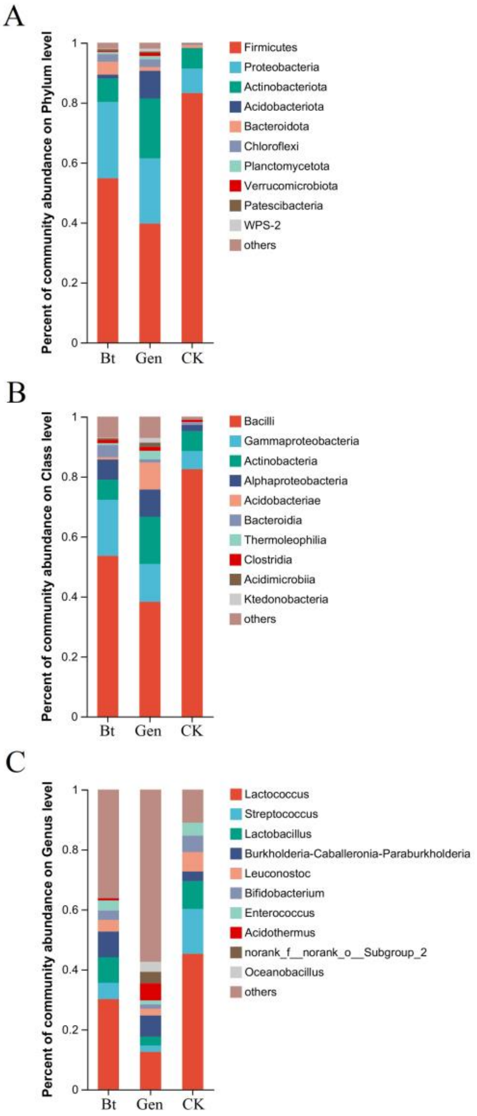

Figure S1 Community composition of intestinal bacteria under different treatments. x-axis presents different treatments, y-axis indicates the percentage of community abundance. (A) community composition at the phylum level, (B) community composition at the class level, (C) community composition at the genus level. Bt, feeding with artificial diet and Cry1Ac; Gen, feeding with 60 μg/mL Gentamicin, artificial diet and Cry1Ac; CK, feeding with artificial diet.

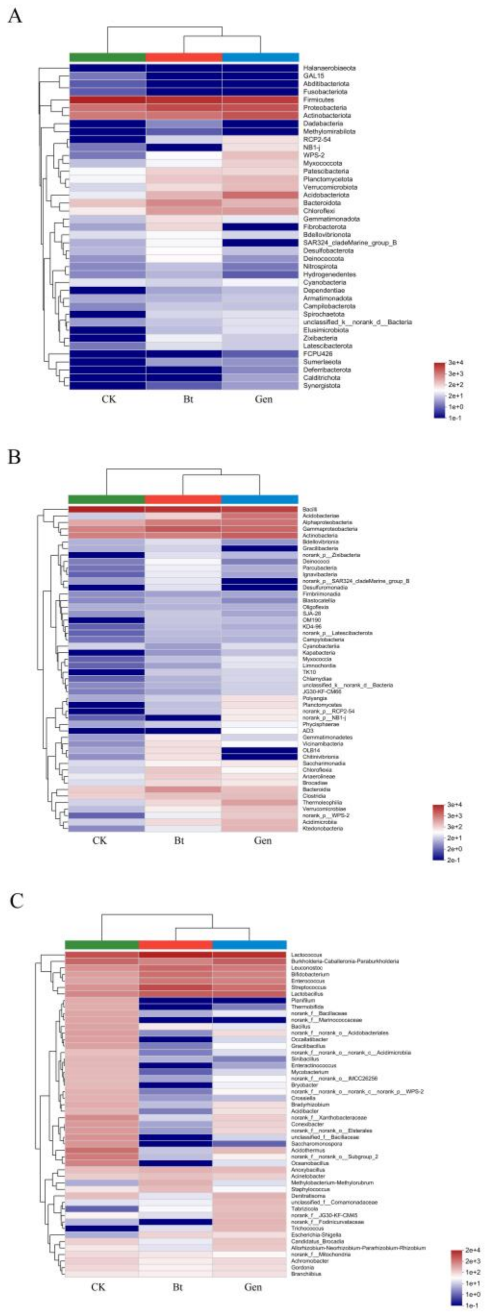

Figure S2 Heatmap analysis under different treatments. x-axis means different treatments. (A) heatmap analysis at the phylum level, (B) heatmap analysis at the class level, (C) heatmap analysis at the genus level. Bt, feeding with artificial diet and Cry1Ac; Gen, feeding with 60 μg/mL Gentamicin, artificial diet and Cry1Ac; CK, feeding with artificial diet.

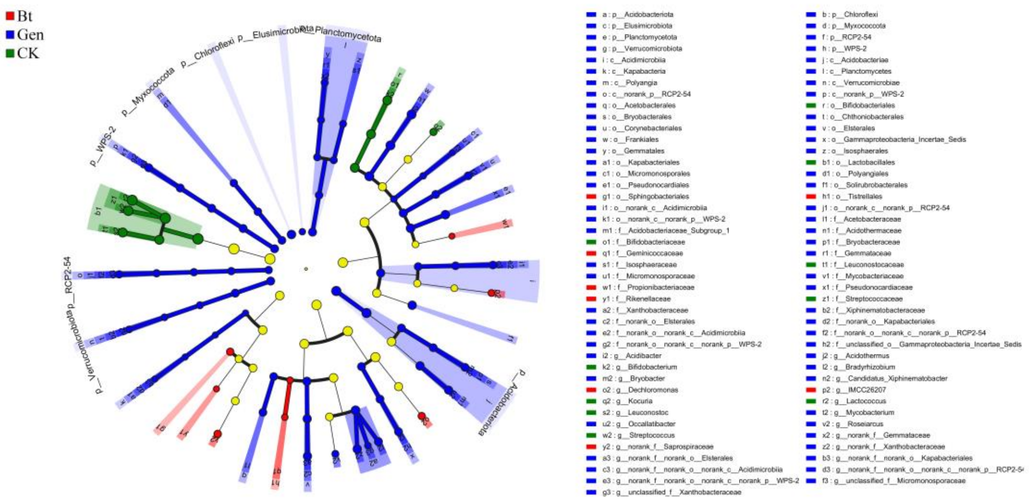

Figure S3 Linear discriminant analysis effect size analysis of the community composition of intestinal bacteria in five taxon units under different treatments. Circles from the inside to the outside represent taxonomy from phylum, class, order, family to genus, respectively. Bt, feeding with artificial diet and Cry1Ac; Gen, feeding with 60 μg/mL Gentamicin, artificial diet and Cry1Ac; CK, feeding with artificial diet.
